# Supplementary material for: Proton conductance by human uncoupling protein 1 is inhibited by purine and pyrimidine nucleotides
Source: EMBO J. 2025 Feb 28;44(8):2353–65. doi: 10.1038/s44318-025-00395-3 (PMC12000319; doi:10.1038/s44318-025-00395-3)
Supplement: Supplementary file 1 — Appendix [file 44318_2025_395_MOESM1_ESM.pdf]

Appendix for Proton conductance by human uncoupling protein 1 is inhibited by purine and pyrimidine nucleotides

Appendix Figure S1 2
Appendix Figure S2 3
Appendix Figure S3 4
Appendix Figure S4 5
Appendix Figure S5 6
Appendix Figure S6 7
Appendix Figure S7 8
Appendix Table S1 9

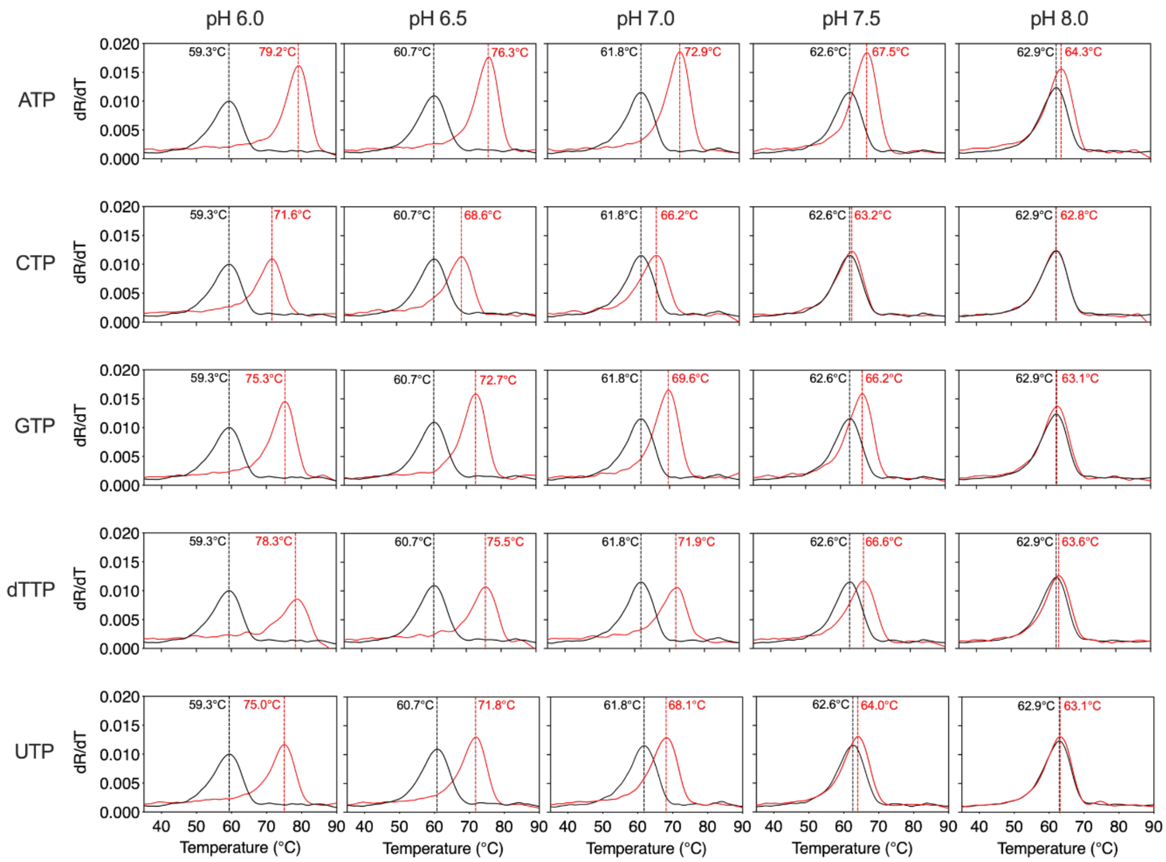

Appendix Figure S1. Nucleotide binding to UCP1 is pH dependent. Representative curves showing the first derivative of the unfolding curves of UCP1 with (red) and without (black) 1 mM nucleotides at pH range 6.0 – 8.0. Dotted lines show the apparent melting temperature (T_m).

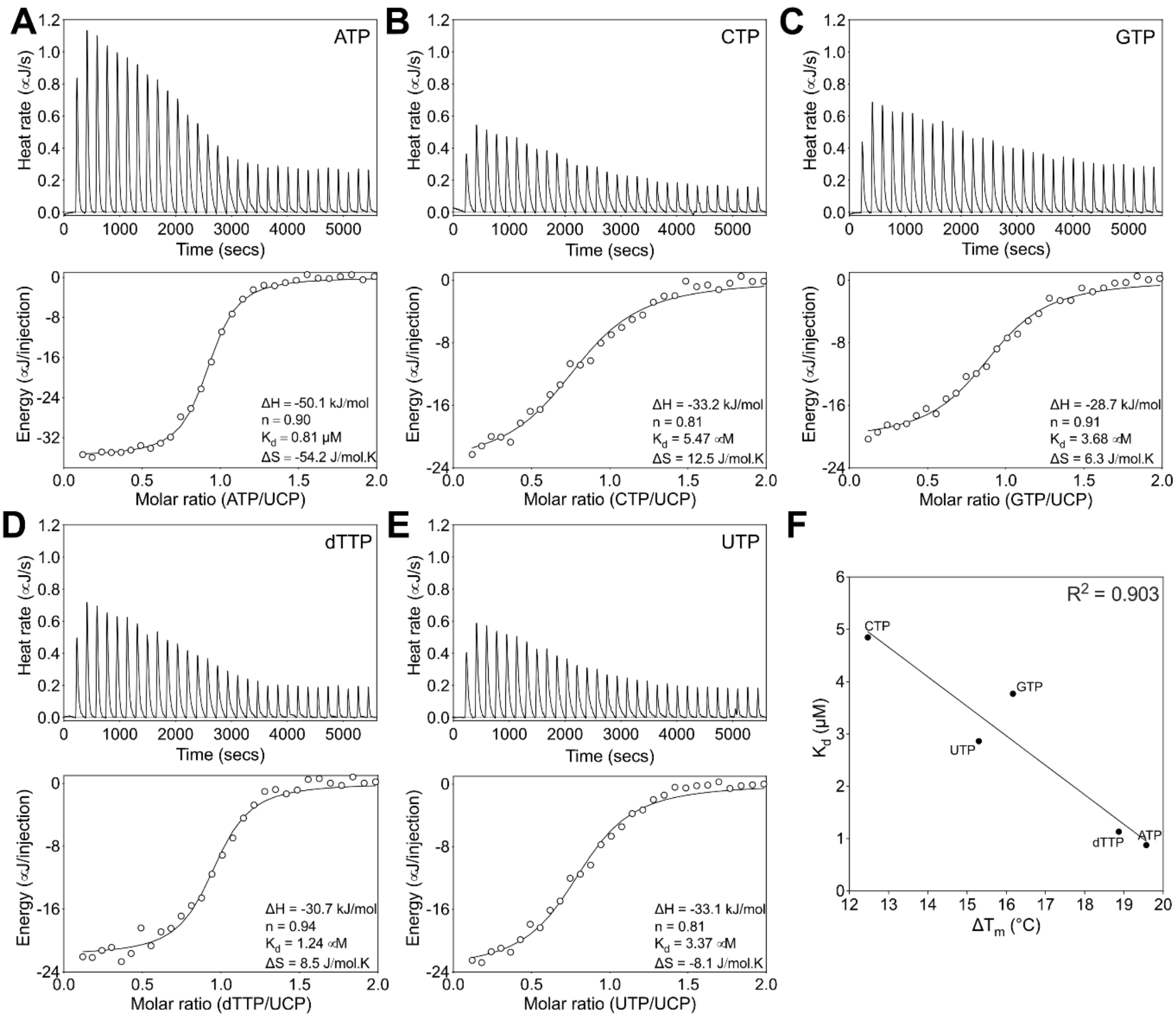

Appendix Figure S2. Nucleotide binding to UCP1 measured by isothermal calorimetry. Representative traces from one repeat showing the enthalpy change caused by nucleotide binding for each 1 µL injection of 750 µM nucleotide (top) and isotherms fitted to a one-site binding model with ΔH, K_d, and stoichiometry as fitting parameters (bottom) with each tested nucleotide **A** ATP, **B** CTP, **C** GTP, **D** dTTP and **E** UTP. **F** Correlation between thermal shift (ΔT_m) caused by 1 mM nucleotide at pH 6.0 measured by differential scanning fluorimetry versus the K_d measured by isothermal calorimetry.

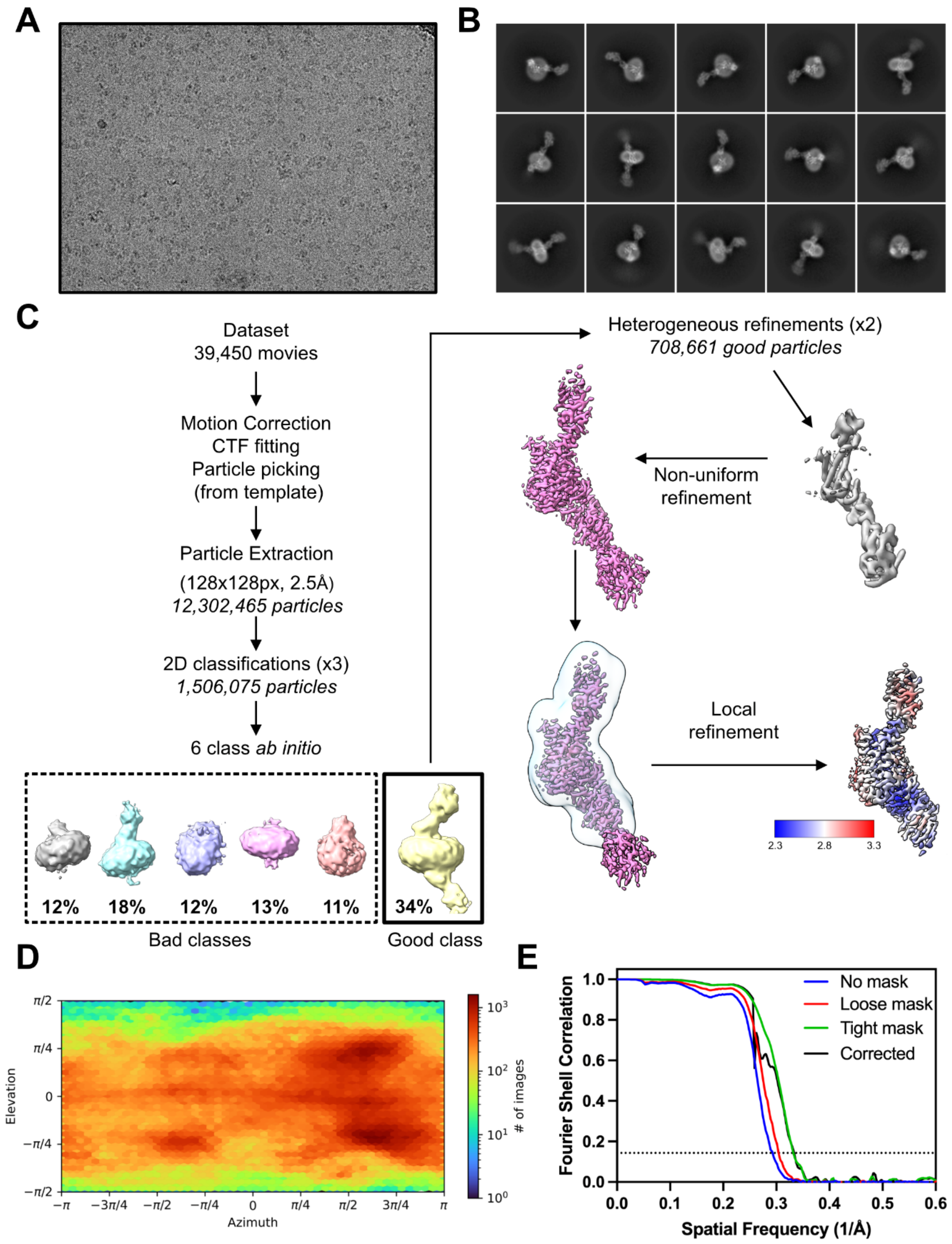

Appendix Figure S3. Cryo-EM data processing. **A** Example micrograph of UCP1-PMb71-PMb65 complex with 2 mM UTP. **B** Example 2D class averages of the complex. **C** Cryo-EM processing workflow to generate final reconstruction. **D** Angular distribution of final reconstruction. **E** Gold standard Fourier Shell Correlation of the non-uniform reconstruction.

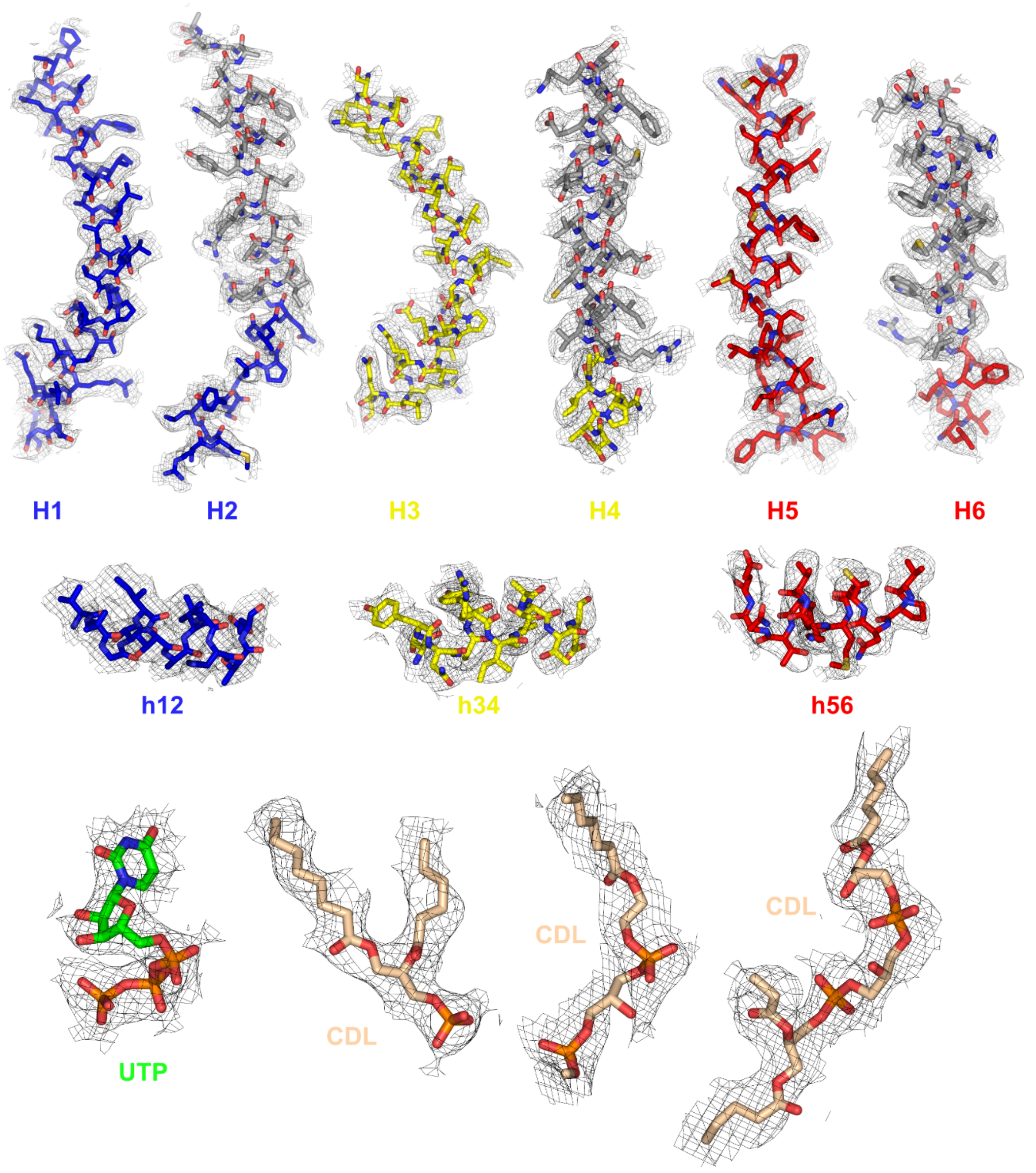

Appendix Figure S4. Cryo-EM electron density. Residues and ligands coloured as described in Fig. 1. Figure prepared using contour Chimera level 0.03 and carve level 2.

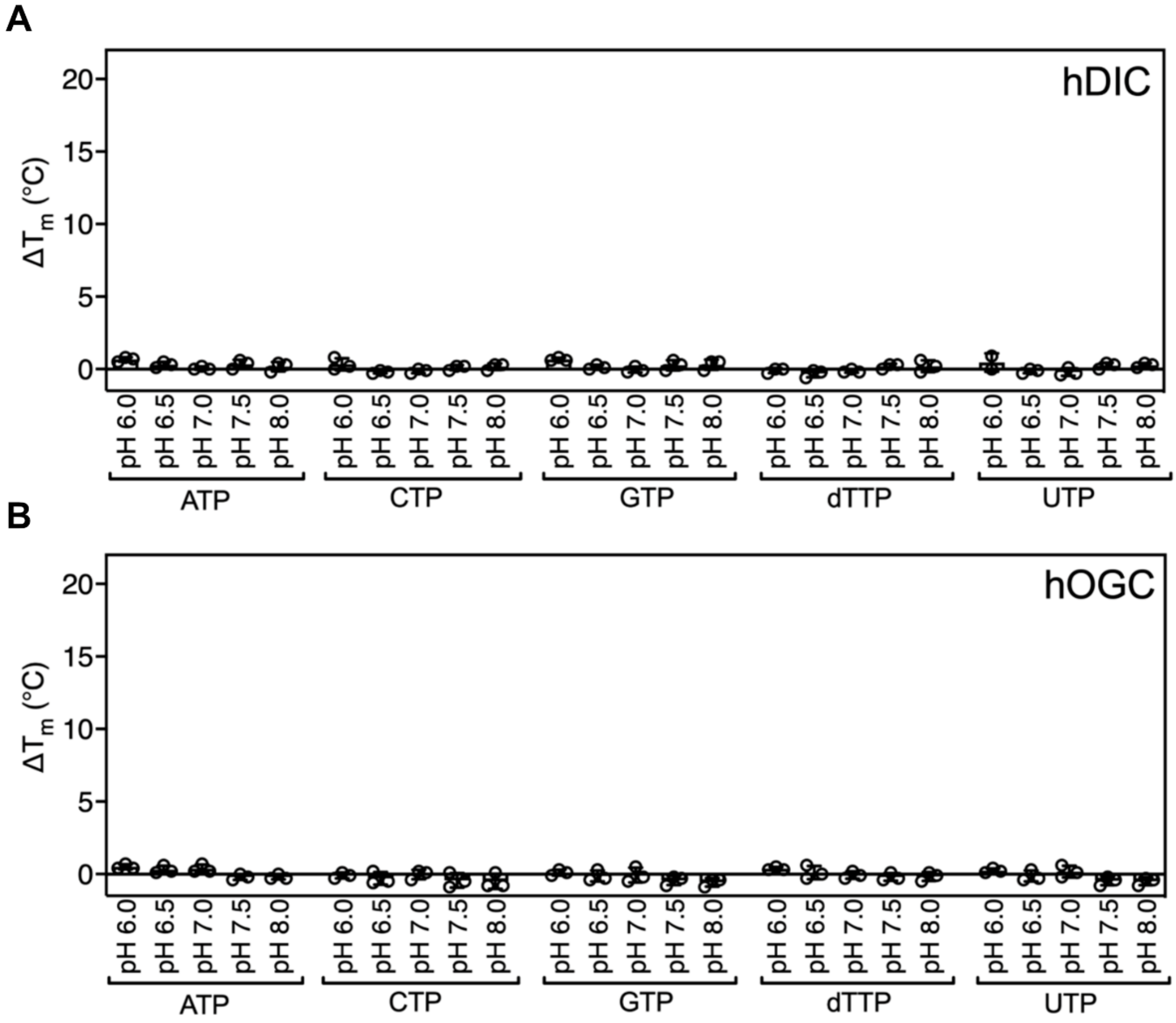

Appendix Figure S5. Effect of nucleotides on the thermostability of hDIC and hOGC. A Change in apparent melting temperature (ΔT_m) of hUCP1, hDIC and hOGC with the addition of 1 mM nucleotide at pH 6.0. **B** ΔT_m with addition of 1 mM nucleotide to purified hDIC at pH range 6.0 – 8.0. **c** ΔT_m with addition of 1 mM nucleotide to purified hOGC at pH range 6.0 – 8.0.

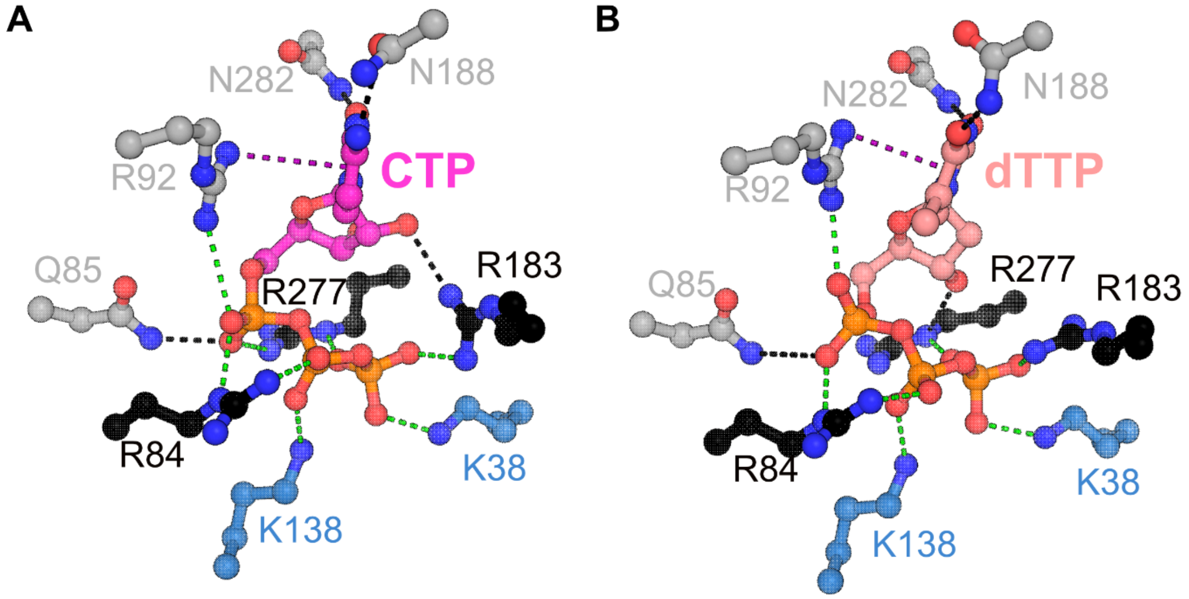

Appendix Figure S6. Modelling of CTP and dTTP binding to UCP1. Modelled using UTP-bound UCP1 structure cryo-EM density showing both nucleotides can bind into the UCP1 nucleotide binding pocket.

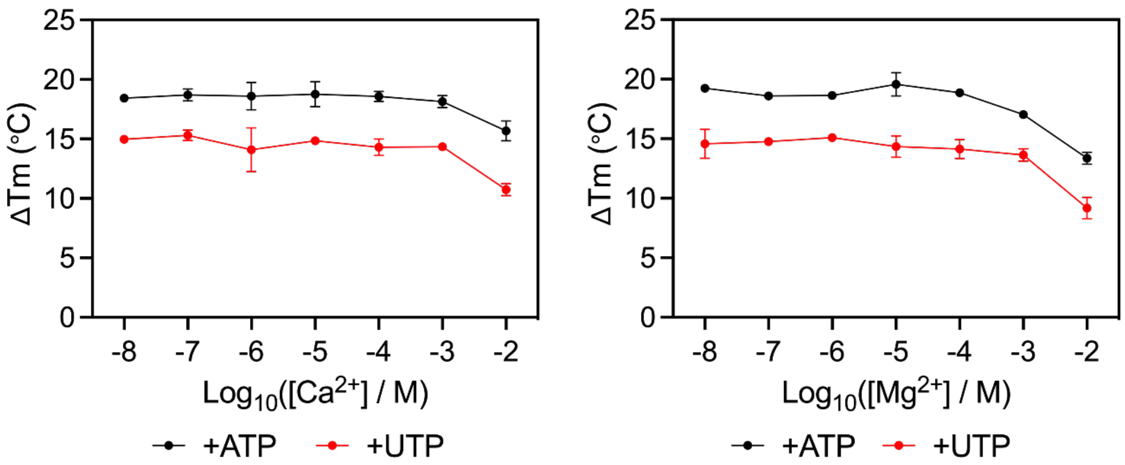

Appendix Figure S7. Effect of Ca^{2+} and Mg^{2+} on UCP1 binding to nucleotides. Binding of either ATP (black) or UTP (red) to human UCP1 measured by thermal stability shift assays. The mean and standard deviation of three biological repeats of the thermal shift (ΔTm) caused by 1 mM nucleotide at pH 6.0 in the presence of a range of Ca^{2+} (left) or Mg^{2+} (right) concentrations.

Appendix Table S1. Cryo-EM data collection, refinement and validation statistics.

	UCP1-PMb71-PMb65 complex (PBD 9FZQ) (EMD-50894)
**Data collection and processing**	
Magnification	130,000
Voltage (kV)	300
Electron exposure (e^-/Å^2)	50
Defocus range (µm)	-1.8 to -0.6
Pixel Size (Å)	0.645
Symmetry imposed	C1
Initial particle images (no.)	12,302,465
Final particle images (no.)	708,661
Map resolution (Å)	3.03
FSC threshold	0.143
**Refinement**	
Initial model used (PDB)	8G8W
Map sharpening B factor (Å^2)	-192.3
**Model composition**	
Non-hydrogen atoms	4088
Protein residues	540
Ligands	UTP:1
	CDL:3
B factors (Å^2)	
Protein	58.33
Ligand	45.19
R.m.s. deviations	
Bond lengths (Å)	0.007
Bond angles (°)	0.794
**Validation**	
MolProbity score	1.06
Clashscore	2.75
Poor rotamers (%)	0.26
**Ramachandran plot**	
Favoured (%)	99.25
Allowed (%)	0.75
Disallowed (%)	0.00
